# Supplementary figures and images for: Dynamic modulation of subthalamic nucleus activity facilitates adaptive behavior
Source: PLoS Biol. 2023 Jun 1;21(6):e3002140. doi: 10.1371/journal.pbio.3002140 (PMC10234560; doi:10.1371/journal.pbio.3002140)

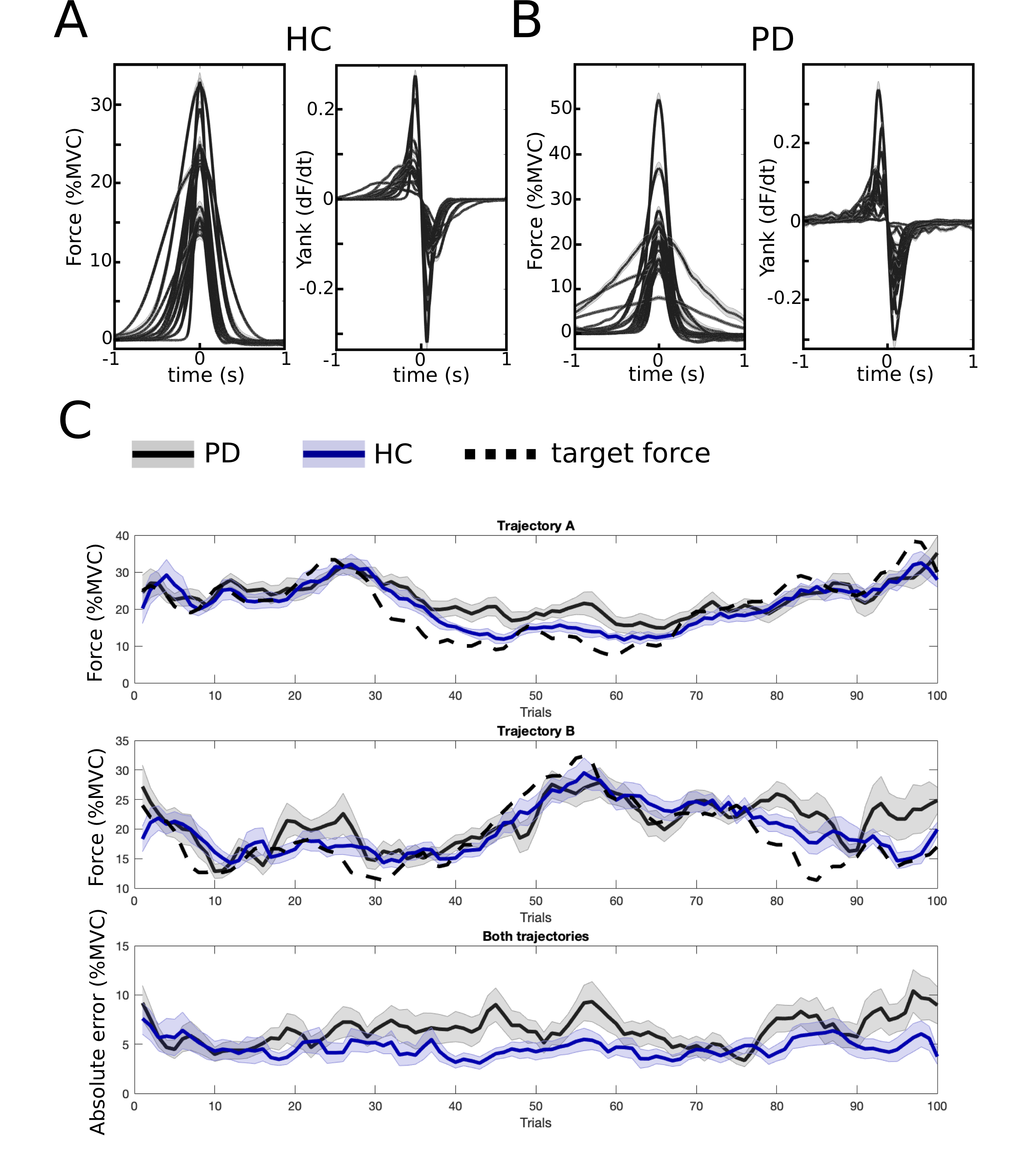

Supplement: S1 Fig — (A) Single participant traces of force and yank (first derivate of force) aligned to peak force for HCs. (B) Same as A but for PD patients. (C) Mean actual force (black: PD patients; blue: HC) are plotted along with target force (dotted lines) for all trials of trajectory A (upper panel) and trajectory B (middle panel). Participants showed the strongest errors when the target force was very low (e.g., middle trials of trajectory A or last trials of trajectory B). This was most pronounced in PD patients as can be seen in the lower panel showing the absolute error over trials. MVC, maximum voluntary contraction. Shaded areas in A–C represent SEM. Underlying data can be found in Mat1 and Mat23 in S1 Data. (TIFF) [file pbio.3002140.s001.tiff]

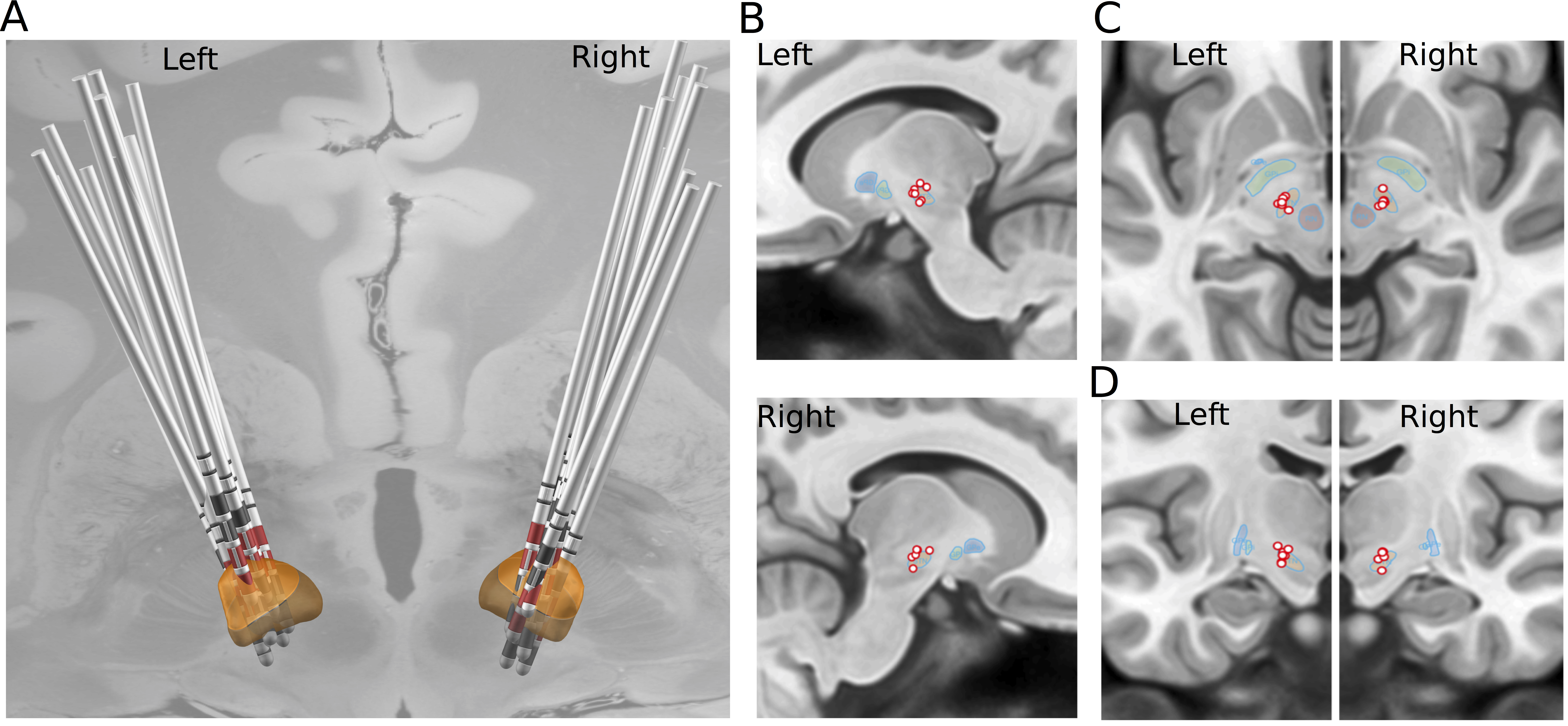

Supplement: S2 Fig — (A) 3D view of reconstructed bilateral leads overlaid on an anatomical mask of the subthalamic nucleus (STN in orange color; the shading is due to the overlay with the axial brain slice). (B–D) Same as A in 2D space separately for sagittal (B), axial (C), and coronal (D) slices. Throughout the figure electrodes from which bipolar LFP signals were analyzed are marked in red (in A some are overlaid by others and therefore obscured). In 4 of 26 hemispheres, both contacts used for the bipolar montage were localized outside the anatomical STN mask according to lead reconstruction (see also S1 Table and Methods). In B–D, STN is indicated by an orange mask, while blue, green, and purple masks indicate, respectively, external pallidum, internal pallidum, and red nucleus. (TIFF) [file pbio.3002140.s002.tiff]

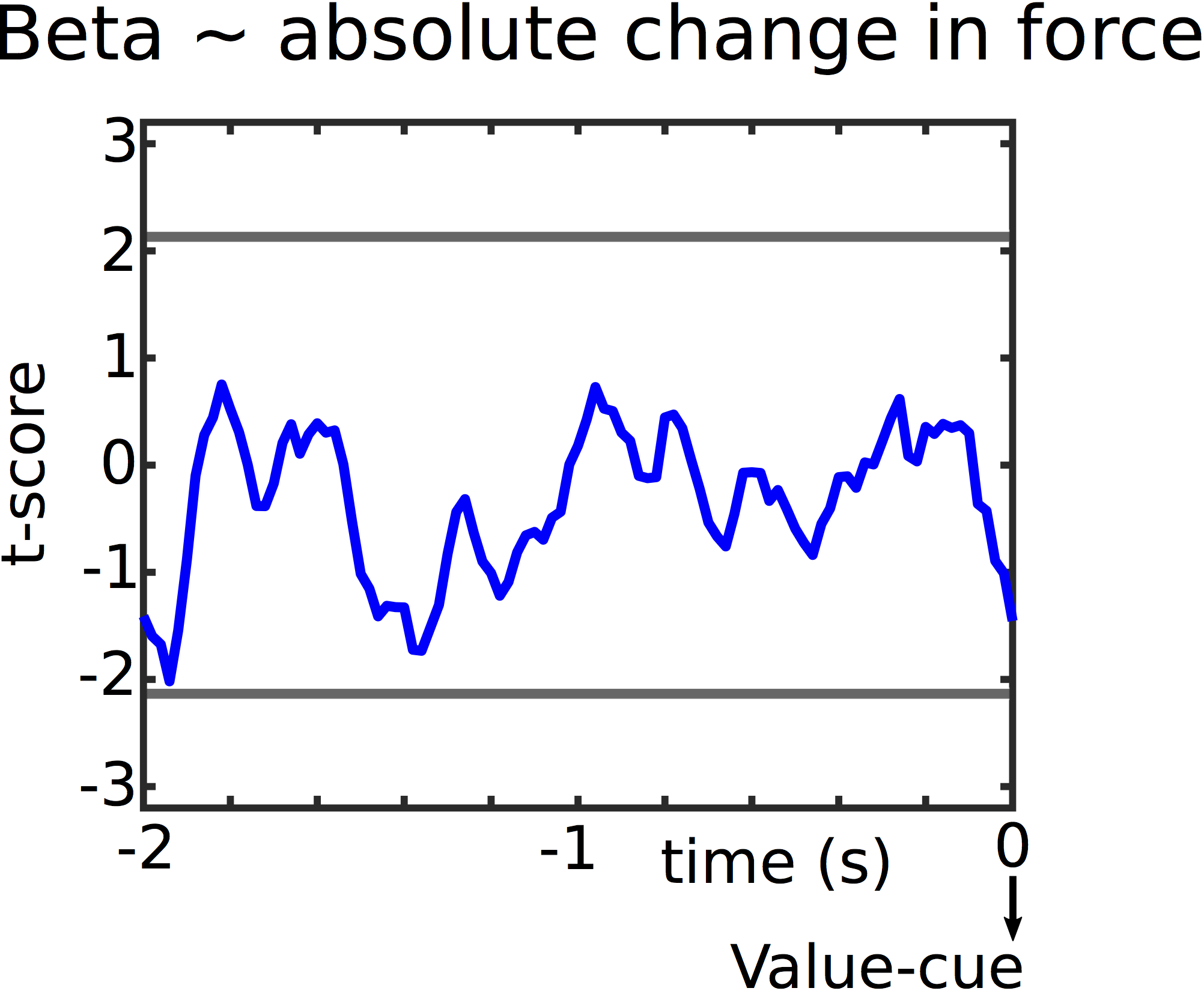

Supplement: S3 Fig — As expected from the varying onsets of movement execution after the Go signal, the relationship between STN beta power and absolute change in force was not present when locking the data to onset of the Value-cue (shown for the 2 s window prior to onset of the Value-feedback). Underlying data can be found in Mat24 in S1 Data. (TIFF) [file pbio.3002140.s003.tiff]

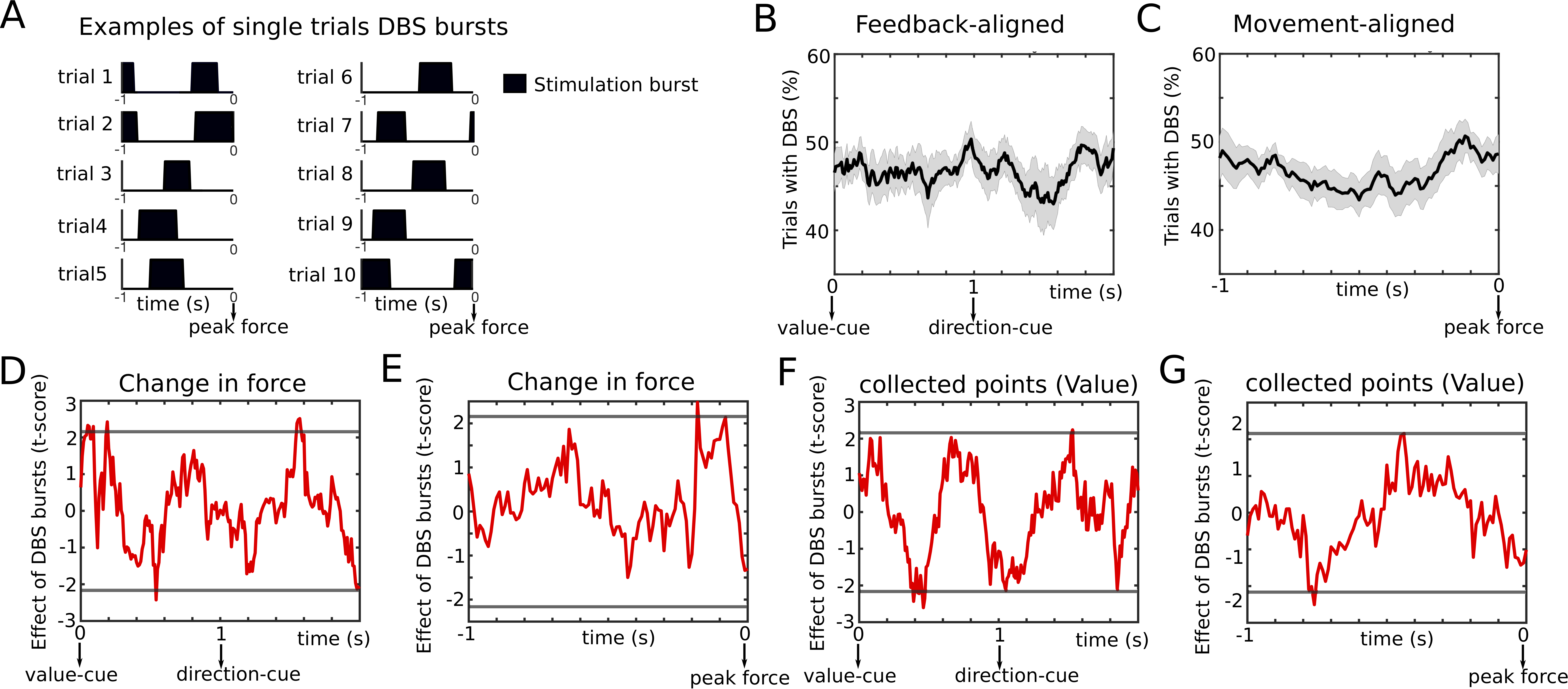

Supplement: S4 Fig — (A) DBS bursts from example trials illustrating when stimulation bursts (black rectangles) were applied in the first 10 trials of patient 1, aligned to peak force. In all patients, DBS was given in bursts of varying duration (mean 250 ms). Between stimulation bursts was an approximately 500-ms long pause (mean 150 ms interval + ramping up and down each lasting ca. 175 ms on average = 500 ms, see also S1 Table). (B) Stimulation occurred on approximately 50% of trials for any given 100 ms window throughout the feedback time period. (C) Same as A for peak force aligned data. (D) There were no significant effects of stimulation on change in force (positive values for increase in force, negative values for decrease in force) on the next trial when aligning data to the feedback cues. (E) Same as D for peak force aligned data. (F) There were no significant effects of stimulation on collected points (Value-feedback) on the next trial when aligning data to the feedback cues. (G) Same as F for peak force aligned data. Shaded areas in B and C represent SEM. Horizontal gray lines in D–G show the cluster-building threshold. DBS, deep brain stimulation. Underlying data can be found in Mat25-30 in S1 Data. (TIFF) [file pbio.3002140.s004.tiff]

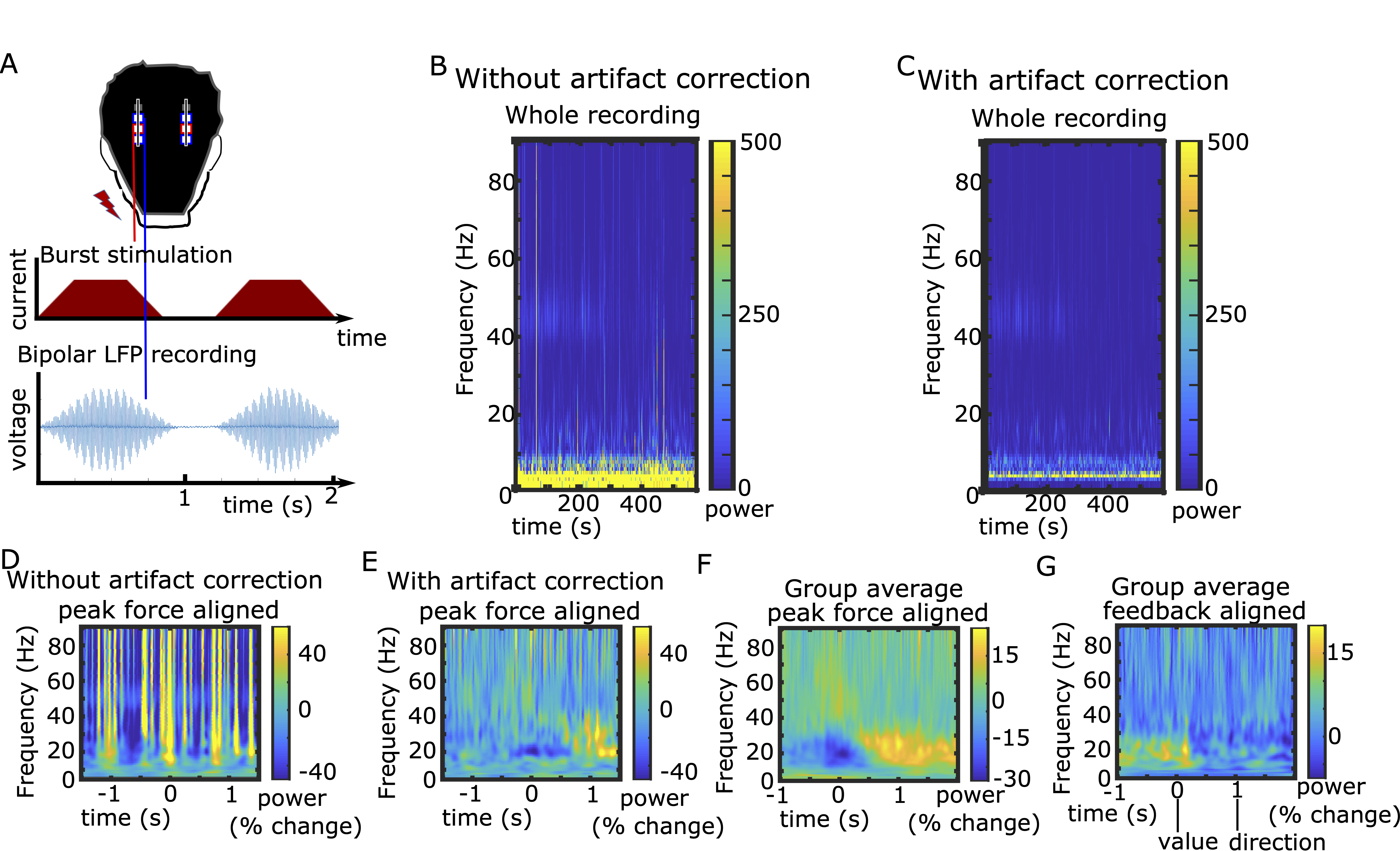

Supplement: S5 Fig — (A) During the stimulation session, local field potentials were recorded from bipolar contacts surrounding the stimulation electrode. Despite common mode rejection, the artifact was clearly visible in the unprocessed data. (B) Example of time-frequency spectrum without artifact correction (see Methods for details regarding artifact correction) for an example patient. The spectral properties of stimulation-related artifacts were not restricted to the stimulation frequency and its harmonics. (C) Same as B but after artifact correction. (D) When the artifacts were strongly expressed, as in this patient (from B), they obliterated the normal movement-related beta power modulation in the trial averaged data (peak force aligned). (E) After artifact correction, the normal (i.e., as observed in the off stimulation session) beta modulation can be seen in the trial averaged data (peak force aligned). (F) Group average of peak force aligned spectrum after artifact correction (compare to Fig 3A). (G) Group average of feedback aligned spectrum after artifact correction (compare to Fig 2A). LFP, local field potential. Underlying data can be found in Mat31-34 in S1 Data. (TIFF) [file pbio.3002140.s005.tiff]

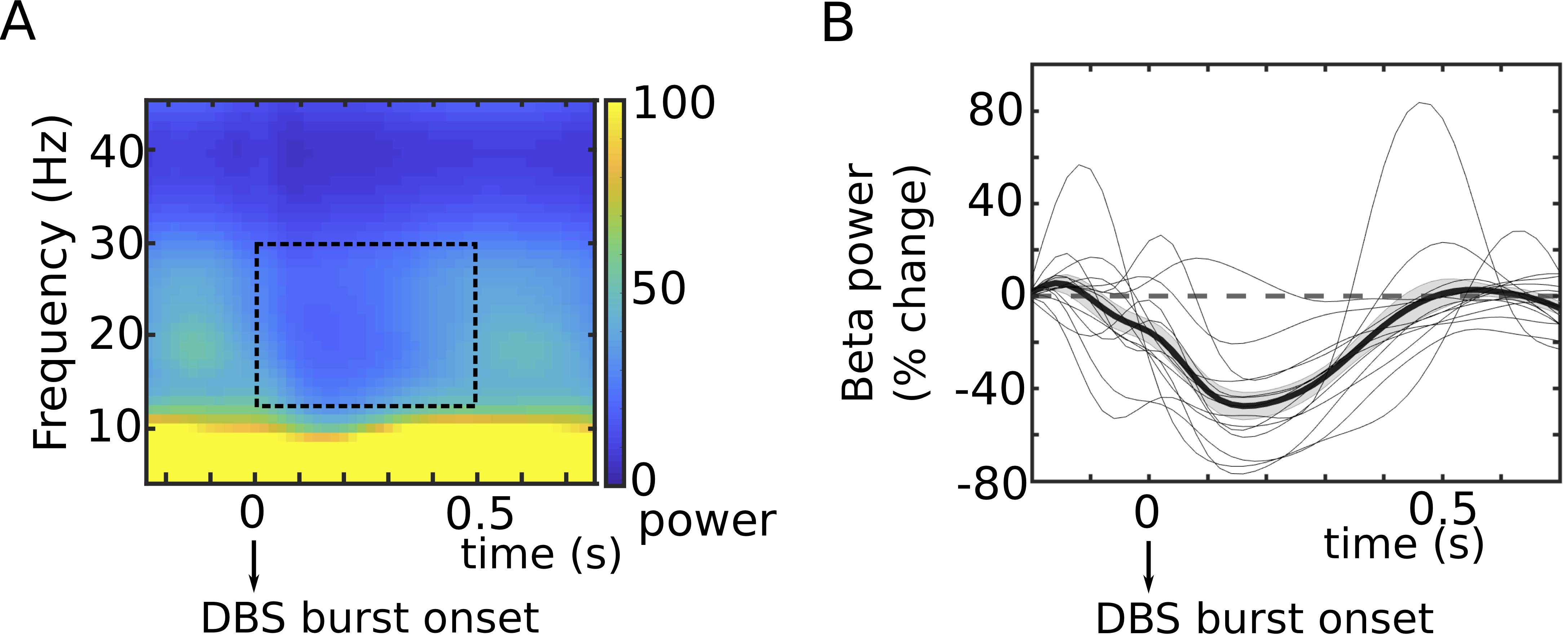

Supplement: S6 Fig — (A) Group-averaged time-frequency spectrum (not normalized) aligned to onset of stimulation (after ramping). The dotted box indicates STN beta power after stimulation onset. For statistical tests of changes in different frequency bands, see S3 Table. (B) Beta power (extracted from dotted box in A) is aligned to onset of stimulation and normalized to the time period where no stimulation was applied. Stimulation led to an approximately 40% decrease in beta power, which returned to baseline after approximately 0.5 s (mean burst duration was 250 ms +/− 100 drawn from a uniform distribution). The thick black line indicates the group mean, and the shaded area around this line represent SEM. The thin gray lines illustrate beta band changes from individual participants. DBS, deep brain stimulation. Underlying data can be found in Mat35-36 in S1 Data. (TIFF) [file pbio.3002140.s006.tiff]

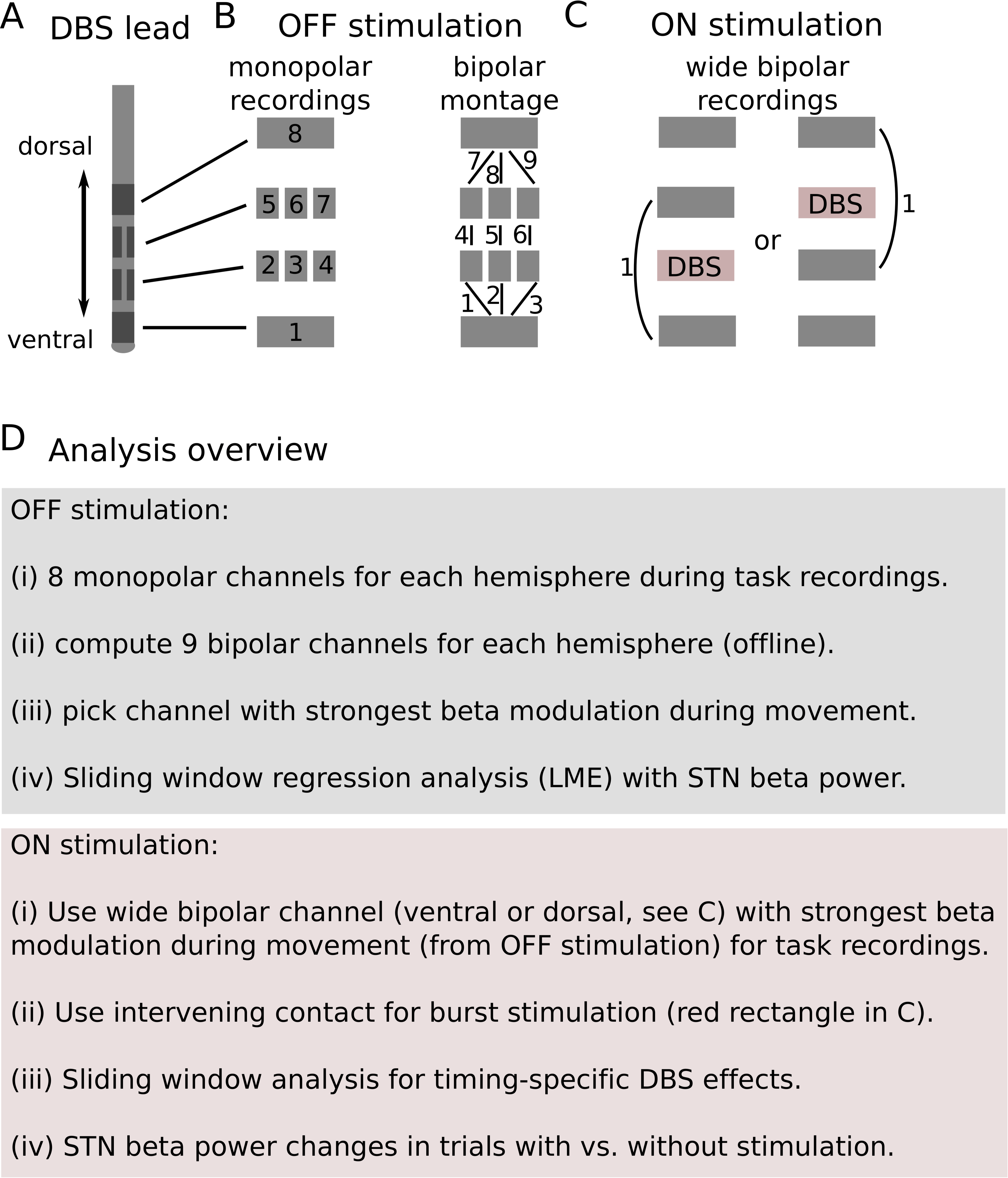

Supplement: S7 Fig — (A) Each DBS (deep brain stimulation) lead had 8 contacts (2 omnidirectional leads and 6 directional leads) on 4 vertical levels. (B) Nine bipolar pairs were computed between neighboring contacts on the vertical levels for the OFF stimulation session. (C) For the ON stimulation session, wide bipolar contacts were created and the intervening contact was used for applying burst stimulation. (D) Analysis overview: For the OFF stimulation session, 9 bipolar contacts per hemisphere were computed based on the 8 monopolar recording contacts. In each hemisphere, the bipolar pair with the most pronounced beta power changes during movement was chosen for further analyses, since this indicates proximity to or localization within dorsal STN and correlates with motor performance (see Methods) and averaged across hemispheres. Single trial values were extracted and regressed against behavioral variables of interest using LME models. During the ON stimulation condition, a wide bipolar contact (shown in C) was created in each hemisphere to mitigate stimulation artifacts using common-mode rejection. The wide bipolar pair showing the most pronounced beta power changes during movement (OFF stimulation) was chosen for recordings. Timing-specific DBS effects on behavior were assessed by analyzing force adaptation in time windows where stimulation was applied vs. not applied in a sliding-window approach. Corresponding DBS effects on STN activity were analyzed for the surrounding bipolar contacts. (TIFF) [file pbio.3002140.s007.tiff]
