# Supplementary material for: Dynamic modulation of subthalamic nucleus activity facilitates adaptive behavior
Source: PLoS Biol. 2023 Jun 1;21(6):e3002140. doi: 10.1371/journal.pbio.3002140 (PMC10234560; doi:10.1371/journal.pbio.3002140)
Supplement: S1 Table — Age and disease duration are given in years. Clinical scores are given as total score of the MDS Unified Parkinson’s disease rating scale (UPDRS) part III for levodopa ON/OFF and as items 3–8 and 14–18 (limb scores) for DBS ON/OFF. Medication is given in levodopa-equivalent daily dose (LEDD). All patients received levodopa, 12 patients received a dopamine-agonist, 10 patients a catechol-O-methyltransferase (COMT) inhibitor, 9 patients a monoamine-oxidase (MAO) inhibitor, and 4 patients amantadine. D and V indicate whether the respectively more dorsal or ventral contact was chosen as active contact in the left and right hemisphere, after which DBS intensity is given in mA and ramp time is given in seconds. The last column lists for each hemisphere whether at least one of the contacts of the bipolar montage used for the local field potential analysis overlaid with a structural mask of the STN (see Methods). n/a, not available. (DOCX) [file pbio.3002140.s008.docx]

| # | Age & gender | UPDRS-III OFF/ON levodopa | UPDRS-III limb OFF/ON DBS | Disease duration | Main symptom | Reason for surgery | Medication (LEDD) | DBS parameters Left / Right | Recording contacts within STN (Left/Right) |
| --- | --- | --- | --- | --- | --- | --- | --- | --- | --- |
| 1 | 60 male | 33 / 14 | 22 / 17 | 6 | Bradykinesia | ON-OFF fluctuations | 1569 mg | D-D, 1.7 mA, 0.215 s | yes / yes |
| 2 | 69 female | 25 / 17 | 24 / 20 | 7 | Bradykinesia | Wearing OFF | 1285 mg | D-V, 2.4 mA, 0.184 s | n/a |
| 3 | 62 male | 32 / 24 | 10 / 4 | 5 | Bradykinesia and tremor | Dyskinesia | 400 mg | D-D, 2.2 mA, 0.169 s | yes / yes |
| 4 | 79 male | 28 / 12 | n/a | 16 | Bradykinesia | ON-OFF fluctuations | 1170 mg | n/a | n/a |
| 5 | 68 male | 59 / 50 | 32 / 29 | 13 | Bradykinesia | ON-OFF fluctuations | 1448 mg | D-D, 2.6 mA, 0.200 s | no / yes |
| 6 | 70 male | 46 / 31 | 23 / 13 | 13 | Bradykinesia | Dyskinesia | 1548 mg | V-V, 1.3 mA, 0.200 s | no / yes |
| 7 | 67 male | n/a | n/a | 19 | Tremor | Wearing OFF | 1480 mg | n/a | yes / yes |
| 8 | 78 male | 44 / 20 | 23 / 13 | 16 | Bradykinesia | ON-OFF fluctuations | 1000 mg | D-D, 2.5 mA, 0.153 s | yes / yes |
| 9 | 71 male | 34 / 32 | 27 / 21 | 4 | Tremor | Tremor | 450 mg | D-D, 2.0 mA, 0.153 s | yes / yes |
| 10 | 68 male | 63 / 33 | 37 / 31 | 30 | Bradykinesia | ON-OFF fluctuations | 892 mg | D-D, 1.1 mA, 0.169 s | no / yes |
| 11 | 73 female | 45 / 26 | 27 / 27 | 11 | Tremor | Tremor | 900 mg | V-V, 2.0 mA, 0.153 s | yes / yes |
| 12 | 73 male | 42 / 23 | 32 / 26 | 6 | Bradykinesia | Gait difficulties | 1514 mg | D-D, 2.5 mA, 0.192 s | n/a |
| 13 | 49 male | 24 / 9 | 36 / 20 | 10 | Bradykinesia | Dyskinesia | 455 mg | D-D, 2.0 mA, 0.153 s | yes / yes |
| 14 | 80 male | 62 / 54 | 39 / 27 | 15 | Tremor | Tremor | 705 mg | D-D, 3.0 mA, 0.230 s | yes / no |
| 15 | 66 male | 23 / 18 | 16 / 13 | 17 | Bradykinesia | ON-OFF fluctuations | 1863 mg | D-D, 1.5 mA, 0.115 s | yes / yes |
| 16 | 25 male | 54 / 32 | 36 / 32 | 3 | Bradykinesia | Dyskinesia | 1065 mg | D-D, 1.6 mA, 0.123 s | yes / yes |
