# Supplementary material for: Dynamic modulation of subthalamic nucleus activity facilitates adaptive behavior
Source: PLoS Biol. 2023 Jun 1;21(6):e3002140. doi: 10.1371/journal.pbio.3002140 (PMC10234560; doi:10.1371/journal.pbio.3002140)
Supplement: S2 Table — au, arbitrary units; AUC, area under the curve; CV, coefficient of variation; dof, degrees of freedom; dt, time derivative; ms, millisecond; MVC, maximum voluntary contraction; N, Newton; RMSE, root mean squared error; s, second. * These 2 effects were significantly different from each other when directly comparing them (t29 = −2.661, d = 0.956, P = 0.013). Significant effects are shown in bold. (DOCX) [file pbio.3002140.s009.docx]

|  | Mean ± standard deviation (PD vs. HC) | t_dof_-value,p-value, Cohen’s d |
| --- | --- | --- |
| Force production: |  |  |
| MVC (N) | 182.3 ± 58 vs. 211.2 ± 60 | t_29_=-1.366, P=0.171, d=0.49 |
| Mean peak force (N) | 40.5 ± 15 vs. 42.9 ± 12 | t_29_= -0.490, P=0.628, d=0.18 |
| Mean peak yank (N/dt) | 0.35 ± 0.1 vs. 0.34 ± 0.1 | t_29_=0.375, P=0.710, d=0.14 |
| Mean peak negative yank (N/dt) | 0.41 ± 0.1 vs. 0.43 ± 0.2 | t_29_=-0.373, P=0.712, d=0.13 |
| AUC (N*ms) | 15212 ± 8003 vs. 15283 ± 7125 | t_29_=-0.026, P=0.979, d=0.01 |
| Time from Go-cue to peak force (s) | 1.13 ± 0.4 vs. 1.05 ± 0.2 | t_29_=-0.645, P=0.524, d=0.23 |
| Peak force–to-peak yank slope | 0.09 ± 0.04 vs. 0.07 ± 0.02 | t_29_=-1.484, P=0.149, d=0.53 |
| Force adaptation: |  |  |
| RMSE (%MVC) | 8.5 ± 2 vs. 6.1 ± 1 | **t_29_=-3.371,** **P=0.002, d=1.21** |
| Average points (au) | 4.9 ± 1 vs. 6.1 ± 1 | **t_29_=3.416,** **P=0.002, d=1.23** |
| Mean force difference (%MVC) | 2.5 ± 3 vs. 0.7 ± 1 | **t_29_=-2.454,** **P=0.020, d=0.88** |
| Actual force during high target force* | 24.7 ± 3 vs. 24.6 ± 2 | t_29_=-0.069, P=0.945, d=0.02 |
| Actual force during low target force* | 20.1 ± 4 vs. 16.6 ± 1 | **t_29_=-3.487, P=0.002, d=1.25** |
| CV (unitless) | 0.33 ± 0.1 vs. 0.35 ± 0.1 | t_29_=0.572, P=0.572, d=0.21 |
| Mean by-trial absolute change in force (%MVC) | 5.3 ± 2 vs. 5.5 ± 2 | t_29_=0.346, P=0.732, d=0.12 |
