# Supplementary material for: Dynamic modulation of subthalamic nucleus activity facilitates adaptive behavior
Source: PLoS Biol. 2023 Jun 1;21(6):e3002140. doi: 10.1371/journal.pbio.3002140 (PMC10234560; doi:10.1371/journal.pbio.3002140)
Supplement: S3 Table — dof, degrees of freedom. Significant effects are shown in bold. (DOCX) [file pbio.3002140.s010.docx]

| Frequency-band: | Change in power after stimulation-onset  (mean ± standard deviation, t_dof_-value, P_corrected_) |
| --- | --- |
| Theta | -9.13 % ± 20.6, t_13_ = -1.597, P = 0.537 |
| Alpha | -13.38 % ± 18.2, t_13_ = -2.647, P = 0.080 |
| **Beta** | **-27.27 % ± 16.7, t_13_ = -5.900, P < 0.001** |
| Gamma | -5.97 % ± 16.1, t_13_ = -1.337, P = 0.817 |
