# Supplementary material for: Dynamic modulation of subthalamic nucleus activity facilitates adaptive behavior
Source: PLoS Biol. 2023 Jun 1;21(6):e3002140. doi: 10.1371/journal.pbio.3002140 (PMC10234560; doi:10.1371/journal.pbio.3002140)
Supplement: S1 Text — (DOCX) [file pbio.3002140.s011.docx]

Patients showed overall poorer task performance compared to healthy controls regarding the difference between actual and target force resulting in lower average Value-feedback (t_29_ = 3.416, d = 1.23, P = 0.002, see Fig 1C and S2 Table for measures of force adaptation). This was mainly due to patients having difficulties in producing very low forces (force trajectories are shown in S1C Fig, the variability across participants in the PD group can also be seen in S1B Fig). Thus, patients produced significantly higher forces compared to HC during low target force trials, while there were no differences between groups in high target force trials (for statistics see S2 Table). However, these difficulties did not impact patients’ overall ability for force adaptation as indicated by similar force level variability (coefficient of variation, t_29_ = 0.572, d = 0.21, P = 0.572) and mean by-trial absolute change in force (t_29_ = 0.346, d = 0.12, P = 0.732) between groups (single participant measures shown in Fig 1D). Importantly, whilst on average overshooting force, patients were still able to reduce their force even when target force levels were low (correlation between force overshoot and decrease in force on the next trial during low target force trials in PD patients: mean rho = 0.364, P < 0.001). This suggests that the group difference in average Value-feedback was due to patients having difficulties in producing very low forces presumably related to impaired dexterity or tremor (an exploratory Pearson correlation between individual tremor scores and force overshoot showed a correlation coefficient of 0.51, P = 0.052) rather than a deficit in force adaptation.
