# Supplementary material for: Dynamic modulation of subthalamic nucleus activity facilitates adaptive behavior
Source: PLoS Biol. 2023 Jun 1;21(6):e3002140. doi: 10.1371/journal.pbio.3002140 (PMC10234560; doi:10.1371/journal.pbio.3002140)
Supplement: Attachment — Submitted filename: ForceAdaptation_ReplyLetter.docx [file pbio.3002140.s013.docx]

**Point-by-point reply to reviewer comments:**

Reviewer #1:

*Herz et al describe findings on action adaptation in 16 PD patients undergoing DBS. Decreases in subthalamic beta (13-30 Hz) activity reflected stronger action adaptation. Moreover, if beta activity was suppressed by STN DBS in a specific time window, this also led to stronger action adaptation suggesting that time-specific modulation of STN beta activity facilitates adaptive behavior.
This is a well-designed and thoroughly conducted study from a highly experienced group. The approach is innovative with the combination of beta activity recording and application of bursts of stimulation to explore the effects of time -specific beta band modulation on behavior. The study shows that dynamics of STN beta activity are related to action adaptation.*

**Reply:** We thank the reviewer for the thoughtful evaluation, which has prompted us to thoroughly revise our manuscript. We hope that we have addressed all concerns as outlined below. Changes in the manuscript are highlighted in red.

*Major points:*

**Comment 1:** *Although the manuscript is well structured, I think it is quite difficult to follow all steps of analysis in the result section and it could be helpful to have a short paragraph that better explains the reasoning for the analysis steps including an overview figure. Moreover, the main result and its value for understanding action adaptation and adjusting DBS should be discussed more specifically.*

**Reply:** We have now included an overview figure as suppl. figure 7D and a short paragraph summarizing the rationale for the analysis in the corresponding figure legend. We have also re-structured the discussion to more clearly describe the main results and their implications for current theories of action adaptation in the basal ganglia (see also replies to comments 3-6 and pages 14-17 in the revised manuscript).

**Supplementary figure 7.** **DBS leads and analysis overview**. **A.** Each DBS (deep brain stimulation) lead had eight contacts (two omnidirectional leads and six directional leads) on four vertical levels. **B.** Nine bipolar pairs were computed between neighboring contacts on the vertical levels for the OFF stimulation session. **C.** For the ON stimulation session wide bipolar contacts were created and the intervening contact was used for applying burst stimulation. **D.** Analysis overview: For the OFF stimulation session 9 bipolar contacts per hemisphere were computed based on the 8 monopolar recording contacts. In each hemisphere the bipolar pair with the most pronounced beta power changes during movement was chosen for further analyses, since this indicates proximity to or localization within dorsal subthalamic nucleus (STN) and correlates with motor performance (see methods), and averaged across hemispheres. Single trial values were extracted and regressed against behavioral variables of interest using linear mixed effects (LME) models. During the ON stimulation condition, a wide bipolar contact (shown in C) was created in each hemisphere to mitigate stimulation artefacts using common-mode rejection. The wide bipolar pair showing the most pronounced beta power changes during movement (OFF stimulation) was chosen for recordings. Timing-specific DBS effects on behavior were assessed by analyzing force adaptation in time windows where stimulation was applied vs. not applied in a sliding-window approach. Corresponding DBS effects on STN activity were analyzed for the surrounding bipolar contacts.

**Comment 2:** *Moreover, the authors state that "this provides further evidence for the usefulness of STN beta activity as a read-out or feedback signal for adaptive DBS approaches and demonstrates that precisely delivered electrical stimulation can modify movement adaptation in humans." In my mind it is far fetched that time specific adaptive DBS could be adjusted to modify action/movement adaptation. The authors do not know from their study if a general (adaptive) beta suppression has an influence on action adaptation or if beta suppression at different time points may even interfere with adaptation.*

**Reply:** We agree that this might have been too speculative and have therefore deleted the sentence regarding adaptive DBS.

**Comment 3:** *The authors point out that they observed a decrease in beta activity after worse outcomes (other than expected with reduced dopamine release). This should be discussed also in the light of the dopaminergic treatment that was used in the study.*

**Reply:** Previous studies have shown that resting STN beta power is elevated in patients with Parkinson’s disease OFF dopamine and this can be reduced by dopamine intake (see [1] for a review). Dopamine intake in PD patients reduces resting STN beta power, but interestingly leads to more pronounced modulation of STN beta power related to cues and movement [2, 3]. In other words, the main interest of the study, i.e. event-related changes in STN beta power, is more accessible to study ON medication. We have included this discussion on page 18 in the revised manuscript. For putative un-physiological effects of dopaminergic medication in patients experiencing medication-induced impulse control disorders, please see reply to comment 5.

**Comment 4:** *I do not follow what they mean by "a more nuanced relationship between dopamine release and beta power".*

**Reply:** We apologize for the unclear phrasing. We meant to say that there is not simply a negative linear relationship between dopamine release and beta power (i.e. high dopamine = low beta), but a more complicated relationship, at least at the time scales relevant for this study, as suggested by work from Graybiel and colleagues [4]. We have clarified this statement in the revised manuscript (page 16).

**Comment 5:** *Additionally, authors have conducted their study ON medication and use this as an argument to have "normal" motor performance in their patients. However, interaction of levodopa treatment and the concept of reward-related dopamine release should be discussed.*

**Reply:** We agree that dopaminergic medication does not necessarily normalize behavior of PD patients. For example, whilst improving movement vigour, dopamine intake can result in abnormal reward processing and learning, which can impair patients’ ability to make decisions and control impulses. This study did not include patients suffering from impulse control disorders. Still, caution should be taken when equating dopamine intake with normalization of function. To assess putative negative effects of dopamine intake on behavioral adaptation, studies testing patients both ON and OFF dopaminergic medication could be conducted. We have now included this important aspect in the revised discussion (page 17).

**Comment 6:** *Moreover, it is known that beta activity is suppressed by levodopa treatment. Shouldn't the effects of STN DBS be more clear with patients OFF medication?*

**Reply:** Interestingly, while dopamine intake in PD patients reduces resting STN beta power, it leads to more pronounced modulation of STN beta power related to cues and movement [2, 3]. As we hoped to link beta activity changes to behavioral changes, and to modify both through DBS, we elected to test subjects ON medication so that beta activity changes would be most apparent. Please also see reply to comment 3.

**Comment 7:** *I also do not follow the rational to argue that recordings immediately after surgery are of specific value. DBS stun effect has been shown to interfere with LFP recordings and that should be mentioned.*

**Reply:** Following the reviewer’s suggestion we have removed the sentences regarding beneficial effects of immediate post-operative recordings from the manuscript and now also mention possible stun effects on the LFP recordings in the revised manuscript (page 19).

**Comment 8:** *BIC differences of the different models are quite similar. Could the authors comment on the relevance of difference of a few points.*

**Reply:** The Bayesian Information Criterion (BIC) is a measure for how well a certain statistical model performs *relative* to other models. Formally it is computed by subtracting the probability of obtaining the observed data given the model (i.e., how well the model performs) from a complexity term (punishing models with more parameters). Thus, a model with lower BIC is considered superior to the model it is compared to. There is no strict significance testing (as in frequentist p-values), but as a commonly accepted guideline a difference of > 2 is considered positive evidence, a difference of >6 is considered strong evidence and a difference >10 is considered very strong evidence [5]. In our analysis the Value-model had a BIC value that was 12 points lower (i.e., very strong evidence) than the Direction-model (page 7) and the Absolute change in force-model had a BIC value that was 8 points lower (i.e., strong evidence) than the change in force-model (page 9). We explain the interpretation of BIC values more clearly in the revised manuscript (page 26).

Reviewer #2:

*First of all, I would like to congratulate the authors on the approach they took and the amount of effort that must have gone into this study. Combining DBS recordings and stimulation within participants is in my view an excellent way to tackle the research questions of this project. I have some open questions, in particular regarding the specificity of the results, which I outline below.*

**Reply:** We thank the reviewer for the positive evaluation and very helpful comments, which we believe have further improved the manuscript. We hope that we have answered all concerns as detailed below. Changes in the manuscript are highlighted in red.

*Major:*

*1. General stimulation effects / lack of sham stimulation condition*

**Comment 1:** *How do the authors exclude general stimulation effects? They compare DBS vs. Off, but how do we know that it is the STN-specific DBS that is driving the observed effects and not a general stimulation effect that could be reached by stimulation anywhere in or even outside the brain? The authors do provide data on the temporal specificity of the stimulation (not all stimulations at all time points result in behavioral changes), but could they provide data on the spatial specificity as well?*

**Reply:** We agree that it is important to rule out unspecific stimulation effects underlying the results.

First, there could be general effects of stimulation on e.g. alertness or effects induced by sensory stimulation (e.g. DBS-induced paresthesia), which relate to the lack of a sham condition mentioned above. There are several findings that argue against such effects. (i) As the reviewer mentions the DBS effects were temporally specific. Stimulation in certain short time windows affected patients’ ability to adapt their force, whilst stimulation in neighboring time windows did not. Since these different stimulation trials occurred in the same session, it is unlikely that they were driven by e.g. fatigue, general changes in alertness or similar. (ii) Any unspecific stimulation effects related to altering e.g. alertness or attentional mechanisms should result in an improvement (or deterioration) of task performance. However, we did not find such general differences in task performance as indicated by the overall number of collected points (pages 10&12). (iii) To avoid paresthesia DBS intensity was carefully titrated (and ramped up and down, which in pilot experiments had shown to mitigate paresthesia). We asked each patient whether they could feel a change in sensation or bursts of paresthesia when titrating stimulation intensity, and if this was the case, DBS intensity was reduced. This aspect is now more clearly described in the revised paper (page 25).

The second aspect refers to spatial specificity. In theory, as pointed out by the reviewer, the same behavioral effects might be induced by stimulating elsewhere in the brain. For DBS experiments, this cannot easily be controlled by a control stimulation site, since stimulation of a DBS electrode localized outside the STN can result in uncomfortable side effects, such as muscle contractions or eye deviations, and given its invasive nature, DBS electrodes cannot additionally be implanted in a ‘control’ nucleus. Nevertheless, given inter-individual variability in the exact placement of DBS electrodes in relation to the STN (figure 6A, see also reply to comment 2.2) and passing fibers, spatial specificity of stimulation effects can be assessed post-hoc using a recently developed approach termed DBS Network Mapping [6]. Here, for each hemisphere the volume of activated tissue around the active contact is computed based on an electric conduction model [7], the individual electrode position and individual stimulation parameters. Next, the stimulation volume is used as seed region for cortical connectivity analysis using a published connectome dataset in PD patients [8, 9] reflecting how strongly the cortical areas are connected to the stimulation volume. Then, Spearman correlation analyses can be performed using this connectivity value and the individual’s behavioral effect of DBS (in our case DBS effects on absolute change in force). Large correlation coefficients indicate that if STN subparts and their afferents connected to a given cortical area were stimulated relatively strongly given the exact localization of the stimulation electrode then the behavioral DBS effect on absolute change in force was also large.

To statistically assess spatial specificity of DBS effects in our study, we defined a cortical area that we *a priori* hypothesized to be related to stronger behavioral stimulation effects, i.e. motor cortex, which has been shown to be centrally involved in force production and adaptation [10-12], is structurally connected to STN (in particular to dorsal STN expressing STN beta activity [13]) and related to clinical improvement after STN-DBS in PD [14]. As a control site, we chose a region that also is structurally connected to the STN [15], but we hypothesized not to be involved in STN DBS effects on force adaptation , i.e. temporal cortex. Thus, we hypothesized that stimulation of STN subparts and afferents connected to motor cortex, but not temporal cortex, should be related to the behavioral effect of DBS. In line with this hypothesis, we found a significant correlation between connectivity of the stimulation volume to motor cortex (rho = 0.62, P_corrected_ = 0.037), but not to temporal cortex (rho = -0.22, P_corrected_ = 0.499), see figure 6B. The motor cortex connectivity correlation was also significantly stronger than the temporal cortex correlation in a direct comparison (z = 2.082, P = 0.037). These results suggest that the behavioral DBS effects indeed showed spatial specificity in that they were more strongly expressed if the stimulation volume included motor cortical connectivity (illustrated in figure 6C). These valuable additional analyses are included on pages 13&14 in the revised manuscript.

**Figure 6.** **Connectivity mapping of DBS effects. A.** 3D view of reconstructed bilateral leads overlaid on an anatomical mask of the subthalamic nucleus (STN; orange color). Active contacts, i.e., electrodes used for deep brain stimulation (DBS), are marked in red showing inter-individual spatial variability. **B.** Results from Spearman rank correlations between connectivity of the stimulation volume to motor cortex (left panel) and temporal cortex (control site, right panel) with inter-individual differences in the behavioral effect of DBS on absolute change in force (mean of DBS_value_ and DBS_move_, see methods). **C.** For illustration purposes, this 3D map of the cerebral cortex shows voxel-wise correlation values between cortical connectivity to stimulation volumes and the behavioral effect of DBS (same variable as in B). Positive values (red color) largely cluster around motor cortical areas. The figure is thresholded at rho = ± 0.1 for illustration purposes only.

*2. Electrode rereferencing and localization*

**Comment 2.1:** *-The re-referencing scheme is quite complex. I am worried that readers may not be familiar with the specific electrode model and its layout with multiple types of contacts. It would be helpful to have a sketch in the supplementary material, depicting an electrode with omni- and directional contacts and how these contacts are rereferenced against each other. It would be great to have sketches for recording contacts as well as for the stimulation contacts, since the referencing scheme was different.*

**Reply:** We have now included a figure (supplementary figure 7A-C; D is shown under reply to comment 1 of reviewer 1) illustrating the DBS contacts and the different reference montages for the OFF and ON stimulation condition.

**Supplementary figure 7.** **DBS leads and analysis overview**. **A.** Each DBS (deep brain stimulation) lead had eight contacts (two omnidirectional leads and six directional leads) on four vertical levels. **B.** Nine bipolar pairs were computed between neighboring contacts on the vertical levels for the OFF stimulation session. **C.** For the ON stimulation session wide bipolar contacts were created and the intervening contact was used for applying burst stimulation.

**Comment 2.2:** *- on pages 20/21, the authors describe that they picked the bipolar pair for further analyses based on movement related decrease in beta power and state that they "confirmed the validity of this functional localizer approach by conducting lead localization analysis", referring to Suppl. Fig. 5, which contains a depiction of the electrodes and an atlas. On p. 15 they say that the contacts "mainly localized to the 'motor' STN." From the figure alone, it is hard to tell whether and which the contacts that were picked via the functional localizer also fell into the STN target region.*

*Could the authors provide this information in a table? Please also add information about inclusion/exclusion rules: e.g., did both or one contact that was merged into bipolar pair have to be in the target region, etc.? Adding another angle/plane to the depiction in Suppl. Fig. 5 might also help to better grasp the localization of the electrodes.*

*Do the main results still hold if the authors only analyze recordings from bipolar pairs that show the largest decrease in beta power and at the same time at least one of the contacts fell into the 'motor' STN?*

**Reply:** We agree that in previous suppl. figure 5 it was difficult to disentangle individual lead reconstructions and active contacts. We have now modified this figure including a 3D overview as well as 2D coronal, sagittal and axial views so that the localization of recording contacts is clearer (see revised suppl. figure 2; stimulation contacts are illustrated in revised figure 6, see above).

Regarding inclusion / exclusion criteria we relied on the electrophysiological instead of a structural localizer, since electrode reconstructions that require a post-operative CT scan were not available at the time of the experiment, and since STN beta power is a good predictor of contact localization and its modulation by movement is related to motor performance [2, 16-18]. Thus, contacts were chosen purely based on electrophysiological properties, not electrode reconstruction. However, we agree that the post-hoc validation of this approach using imaging data is important. Following the reviewer’s suggestion, we now added information regarding the number of chosen bipolar contacts that localized to the STN according to electrode reconstruction on visual inspection (revised manuscript pages 7, 23 & 24, revised suppl. table 1). To this end, for each individual hemisphere we visually inspected the electrode reconstruction and anatomical STN masks and marked whether at least one of the bipolar contacts overlaid with the STN. This was the case for 22 of 26 hemispheres (imaging was not available in 3 patients). To assess whether this affected the LFP analysis, we excluded hemispheres where the contacts were outside the STN from the LFP regression analysis (i.e. instead of the mean signal across hemispheres, only the LFP from the hemisphere with electrode localization within the STN was used). Of note, each patient had a bipolar contact within the STN in at least one hemisphere, i.e. while four hemispheres had to be excluded (contacts outside the STN), no participants had to be excluded for the control analysis. This additional analysis did not alter significance of the results, i.e. the effects of Value (feedback-aligned data, t = 3.206, P = 0.001) and absolute change in force (movement-aligned analysis, t = -2.743, P = 0.006) remained significant. We now report this in the revised manuscript (pages 7&9).

**Supplementary figure 2.** **Localization of recording electrodes. A.** 3D view of reconstructed bilateral leads overlaid on an anatomical mask of the subthalamic nucleus (STN in orange color; the shading is due to the overlay with the axial brain slice). **B-D.** Same as A in 2D space separately for sagittal (B), axial (C) and coronal (D) slices. Throughout the figure electrodes from which bipolar local field potential (LFP) signals were analyzed are marked in red (in A some are overlaid by others and therefore obscured). In 4 of 26 hemispheres both contacts used for the bipolar montage were localized outside the anatomical STN mask according to lead reconstruction (see also suppl. table 1 and methods). In B-D STN is indicated by an orange mask, while blue, green and purple masks indicate, respectively, external pallidum, internal pallidum and red nucleus.

Of note, for these analyses we did not further subdivide STN into separate ‘subterritories’, since motor vs. limbic subareas are unlikely to have clear-cut borders and rather reflect a gradient from dorsolateral (‘motor’) to ventromedial (‘limbic’) STN [19]. The figure clearly shows the leads crossing the dorsolateral part of the STN, as recommended for optimal motor improvement in PD, and sparing the ventromedial tip.

Regarding the relationship between localization of stimulation contacts and behavioral effects of stimulation please see reply to comment 1.

**Comment 2.3:** *-What happens if the stimulation rereferencing scheme is used to build bipolar pairs for the recordings and main analyses are repeated? Do the main results still hold?*

**Reply:** Using the re-referencing from the stimulation session for the no-stimulation session (i.e. wide bipolar recordings, see also reply to comment 2.1) did not alter significance of the results, i.e. the relationship between STN beta power and Value-feedback (t = 2.490, P = 0.013, feedback aligned) as well as absolute change in force (t = -2.286, P = 0.022, movement aligned) remained significant. This is now reported on pages 7&9.

**Comment 3:** *Why do the authors use predefined frequency bands? E.g., alpha peak varies drastically with all kinds of factors, and will also be different in patients and healthy participants. Could the authors use a more data-driven approach to define their frequency bands? This might, for example, increase the signal for gamma power and reveal effects related behavior.*

**Reply:** Following this suggestion we have now also used participant-specific definitions of gamma (based on the individual movement-related gamma increase) and alpha (based on the feedback-related alpha increase) frequency bands for the regression analyses. This did not alter the results, i.e. the regressions with STN alpha and gamma power remained not significant, which is now reported on pages 8&9 in the revised manuscript.

**Comment 4:** *Is it problematic that the onset of the Direction-Cue was predictable? Due to the constant time difference between both cues, some of the processes elicited by the onset of the cue (without having knowledge of the direction) might have shifted in time because they could be anticipated.*

**Reply:** We agree that since the onset of the Direction cue could be predicted, any general responses to the cue could in theory be shifted in time. Thus e.g. the increase in STN alpha power after the Value-cue could also be related to anticipation of the Direction cue. Importantly, however, the content of the Direction cue could not be predicted in line with the lack of a correlation between STN activity and Direction feedback in the 2s long feedback window (comprising Value- and Direction-feedback). We have included a short discussion of this and how it might be avoided in future studies by varying feedback duration in the revised manuscript (page 17).

**Comment 5:** *Why did the authors choose the mean power across the whole recording as a baseline? Would the main results still hold if a baseline within the task was used, e.g., a period prior to value-cue onset?*

**Reply:** To secure a sufficient number of trials, whilst at the same time limiting experiment duration to avoid fatigue, we used a relatively fast design without any prolonged baseline periods. Therefore, we used the mean across the whole recording as a baseline period as we have done in our previous studies (e.g. [20, 21]). We agree that different baseline definitions can affect the results and have therefore conducted an additional analysis in which the baseline was defined as 500 ms before the Value-cue until the Value-cue (this avoids movements occurring during the baseline) as suggested by the reviewer. Importantly, the significant relationship between STN beta power and Value as well as absolute change in force remained significant using this alternative baseline (Value: t = 3.458, P < 0.001, absolute change in force: t = -2.989, P = 0.003, see pages 7&9 in the revised manuscript).

**Comment 6:** *Line 37 & Fig. 5D: Why was this exact time window from 680 to 300 ms pre peak-force chosen? Why not use the time window 440-300 ms pre peak-force as in Fig. 3D? Do the results still hold for the 440-300 ms time window?*

**Reply:** The windows for DBS effects on STN activity (figure 4 & 5) were chosen based on the time windows showing significant relationships between STN beta power and behavior / cue content (400-700 ms post-cue for value-alignment and 460-300 ms before peak force for movement-alignment, figures 2D and 3D) and the time windows with DBS effects on force adaptation (180-340 ms post-cue for value-alignment and 680-580 ms before peak force for movement-alignment, figures 4B and 5B). Thus, the windows of interest were 180-700 ms post Value-cue and 680-300 pre peak-force (shown in figures 4D and 5D). While beta power was lower during stimulation vs. no stimulation until 340 ms pre peak force, the cluster was only significant until 520 ms before peak force. We revised this paragraph to make this more clear (page 13)

*Minor:*

**Comment 7:** *-The authors could indicate the meaning of t=0 in Fig. 1C, in the figure legend.*

**Reply:** T=0 here indicates peak force, which is now included in the figure legend.

**Comment 8:** *-Line 164/165: Was this corrected for multiple comparisons? If yes, please indicate in the text, if no, please do so (comparisons are conducted for multiple time bins, inflating false positives). At the very least, indicate that this is uncorrected.*

**Reply:** General effects of feedback on beta power (i.e. irrespective of cue content) were tested by comparing mean beta power during feedback vs. mean power before the feedback (see methods, lines 769 – 771 in the previous manuscript version), i.e. only one test was conducted for feedback-aligned data. The analogous test was conducted for beta power before compared to during movement (lines 771 – 773 in the previous manuscript version). Multiplying the p-values by 2 (according to Bonferroni) would result in P-values of, respectively, 0.012 and <0.001. On reconsideration, we believe that neither of these analyses is central to our experimental question, which asks whether the extent of STN beta power changes correlates with force adaptation, nor are they novel (STN beta power reductions after cues and during movement have been reported in a multitude of studies). We therefore decided to remove these two statistical tests in order to focus on the regression analyses between by-trial changes in STN activity and behavioral adaptation.

**Comment 8:** *-Please provide more information about the DBS electrodes: How many contacts per lead, how large is a contact, what is the distance between contacts.*

**Reply:** All participants were implanted with bilateral Abbott 6170^TM^ leads. Each lead has 8 contacts on four vertical levels. The uppermost and lowermost contacts are ring contacts while both middle contacts consist of three directional contacts. The height of each contact is 1.5 mm and there is a 0.5 mm space in between the four vertical levels. This is now described in the methods section (page 19).

**Comment 9:** *- Is the effect in beta power locked to peak-force (Fig. 3), also observable when locking the data to the value cue?*

**Reply:** Since movement onsets occurred at different time points after the Go-signal, the movement was not locked to the value-feedback. Thus, we would expect that the beta-force adaptation relationship from the peak-force aligned data should not be present in the feedback-aligned data. This was confirmed in an additional analysis as suggested by the reviewer (see below). We have included this in the revised manuscript (page 9 and suppl. figure 3).

**Supplementary figure 3.** **Relationship between STN beta power and absolute change in force locked to the value cue**. As expected from the varying onsets of movement execution after the Go signal, the relationship between STN beta power and absolute change in force was not present when locking the data to onset of the Value-cue (shown for the 2s window prior to onset of the Value-feedback).

**Comment 10:** *-Are the time-frequency plots in Fig. 2A and 3A informative? Since it is an average across participants, the depicted in-/decreases could be driven by single participants / outliers and potentially be driven by the type of baseline the authors used (mean power across the whole recording can vary drastically across patients). Could the authors add a depiction that takes variability across participants into account?*

**Reply:** Following the reviewer’s suggestion we now show the t-scores instead of the mean power in figures 2A and 3A, since t-scores take into account between-subject variance (the mean is divided by the standard error so that large variance would results in low t-scores). Of note, these spectra are highly similar to the spectra from the previous version. Beta, alpha (feedback-aligned) and gamma (movement-aligned) power changes are also plotted with their standard errors in figures 2B,C and 3B, C. Please see reply to comment 5 regarding different baseline corrections.

**Figure 2.** **Correlates of action evaluation.** **A.** Subthalamic nucleus (STN) time-frequency spectra aligned to the feedback shown as t-scores (mean across participants divided by standard error).

**Figure 3.** **Correlates of action adaptation.** **A.** Subthalamic nucleus (STN) time-frequency spectra aligned to peak force feedback shown as t-scores (mean across participants divided by standard error).

**Comment 11:** *-Looking at the time-frequency plot in Fig. 2A, there seems to be a prominent decrease before the onset of the direction-cue, in a somewhat lower frequency than the alpha increase in Fig. 2B (but see comment above). Do the authors have an idea what is going on there?*

**Reply:** When designing this study we had clear a-priori hypotheses based on previous studies showing changes in STN beta power during force exertion. We also included alpha and gamma power changes in additional analyses, because changes in these frequency bands were clearly visible in the spectra (see figures 2 and 3). It is true that there seem to be other changes (e.g. the ~6-8 Hz change mentioned in this comment, and also smaller increases in lower frequency bands during feedback). We do not have a clear idea about their putative functional roles. We have now conducted an exploratory regression analysis with the ~6-8 Hz change from 600-1100 ms after the Value feedback (based on figure 2A), which did not reveal a significant relationship with Value (t = 0.342, P = 0.732) or Direction (t = 1.019, P = 0.309). We would prefer not to include this additional exploratory analysis in this paper, since this would draw the focus away from the clear hypotheses that we tested in this study and might appear as ‘fishing for significance’ to the reader.

**Comment 12:** *-Line 643: "dorsal STN [31, 32]" vs. line 674: "dorsal STN [13, 31]"
References correct?*

**Reply:** Thank you for spotting this error. Line 674 should indeed cite the same references as line 643. This has been corrected.

**Additional minor correction:** There was a minor error in the calculation of Force in Newton from % maximum voluntary contraction in suppl. table 1. This did not change the results, since it merely affected a constant scaling factor, but has now been corrected.

**References:**

1.	Jenkinson N, Brown P. New insights into the relationship between dopamine, beta oscillations and motor function. Trends Neurosci. 2011;34(12):611-8. Epub 2011/10/25. doi: 10.1016/j.tins.2011.09.003. PubMed PMID: 22018805.

2.	Doyle LM, Kuhn AA, Hariz M, Kupsch A, Schneider GH, Brown P. Levodopa-induced modulation of subthalamic beta oscillations during self-paced movements in patients with Parkinson's disease. Eur J Neurosci. 2005;21(5):1403-12. Epub 2005/04/09. doi: 10.1111/j.1460-9568.2005.03969.x. PubMed PMID: 15813950.

3.	Oswal A, Litvak V, Sauleau P, Brown P. Beta reactivity, prospective facilitation of executive processing, and its dependence on dopaminergic therapy in Parkinson's disease. J Neurosci. 2012;32(29):9909-16. Epub 2012/07/21. doi: 10.1523/JNEUROSCI.0275-12.2012. PubMed PMID: 22815506; PubMed Central PMCID: PMCPMC3752071.

4.	Schwerdt HN, Amemori K, Gibson DJ, Stanwicks LL, Yoshida T, Bichot NP, et al. Dopamine and beta-band oscillations differentially link to striatal value and motor control. Sci Adv. 2020;6(39). Epub 2020/09/27. doi: 10.1126/sciadv.abb9226. PubMed PMID: 32978148; PubMed Central PMCID: PMCPMC7518859.

5.	Raftery AE. Bayesian model selection in social research. Sociological Methodology. 1995;25:111-63. doi: 10.2307/271063.

6.	Neudorfer C, Butenko K, Oxenford S, Rajamani N, Achtzehn J, Goede L, et al. Lead-DBS v3.0: Mapping deep brain stimulation effects to local anatomy and global networks. Neuroimage. 2023;268:119862. Epub 2023/01/08. doi: 10.1016/j.neuroimage.2023.119862. PubMed PMID: 36610682.

7.	Vorwerk J, Oostenveld R, Piastra MC, Magyari L, Wolters CH. The FieldTrip-SimBio pipeline for EEG forward solutions. Biomed Eng Online. 2018;17(1):37. Epub 2018/03/28. doi: 10.1186/s12938-018-0463-y. PubMed PMID: 29580236; PubMed Central PMCID: PMCPMC5870695.

8.	Ewert S, Plettig P, Li N, Chakravarty MM, Collins DL, Herrington TM, et al. Toward defining deep brain stimulation targets in MNI space: A subcortical atlas based on multimodal MRI, histology and structural connectivity. Neuroimage. 2018;170:271-82. Epub 2017/05/26. doi: 10.1016/j.neuroimage.2017.05.015. PubMed PMID: 28536045.

9.	Parkinson Progression Marker I. The Parkinson Progression Marker Initiative (PPMI). Prog Neurobiol. 2011;95(4):629-35. Epub 2011/09/21. doi: 10.1016/j.pneurobio.2011.09.005. PubMed PMID: 21930184; PubMed Central PMCID: PMCPMC9014725.

10.	Cheney PD, Fetz EE. Functional classes of primate corticomotoneuronal cells and their relation to active force. J Neurophysiol. 1980;44(4):773-91. Epub 1980/10/01. doi: 10.1152/jn.1980.44.4.773. PubMed PMID: 6253605.

11.	Cherian A, Fernandes HL, Miller LE. Primary motor cortical discharge during force field adaptation reflects muscle-like dynamics. J Neurophysiol. 2013;110(3):768-83. Epub 2013/05/10. doi: 10.1152/jn.00109.2012. PubMed PMID: 23657285; PubMed Central PMCID: PMCPMC3742991.

12.	Fischer P, Pogosyan A, Green AL, Aziz TZ, Hyam J, Foltynie T, et al. Beta synchrony in the cortico-basal ganglia network during regulation of force control on and off dopamine. Neurobiol Dis. 2019;127:253-63. Epub 2019/03/09. doi: 10.1016/j.nbd.2019.03.004. PubMed PMID: 30849510; PubMed Central PMCID: PMCPMC6517271.

13.	Horn A, Neumann WJ, Degen K, Schneider GH, Kuhn AA. Toward an electrophysiological "sweet spot" for deep brain stimulation in the subthalamic nucleus. Hum Brain Mapp. 2017;38(7):3377-90. Epub 2017/04/09. doi: 10.1002/hbm.23594. PubMed PMID: 28390148; PubMed Central PMCID: PMCPMC6867148.

14.	Horn A, Reich M, Vorwerk J, Li N, Wenzel G, Fang Q, et al. Connectivity Predicts deep brain stimulation outcome in Parkinson disease. Ann Neurol. 2017;82(1):67-78. Epub 2017/06/07. doi: 10.1002/ana.24974. PubMed PMID: 28586141; PubMed Central PMCID: PMCPMC5880678.

15.	Brunenberg EJ, Moeskops P, Backes WH, Pollo C, Cammoun L, Vilanova A, et al. Structural and resting state functional connectivity of the subthalamic nucleus: identification of motor STN parts and the hyperdirect pathway. PLoS One. 2012;7(6):e39061. Epub 2012/07/07. doi: 10.1371/journal.pone.0039061. PubMed PMID: 22768059; PubMed Central PMCID: PMCPMC3387169.

16.	Devos D, Szurhaj W, Reyns N, Labyt E, Houdayer E, Bourriez JL, et al. Predominance of the contralateral movement-related activity in the subthalamo-cortical loop. Clin Neurophysiol. 2006;117(10):2315-27. Epub 2006/08/24. doi: 10.1016/j.clinph.2006.06.719. PubMed PMID: 16926112.

17.	Tamir I, Wang D, Chen W, Ostrem JL, Starr PA, de Hemptinne C. Eight cylindrical contact lead recordings in the subthalamic region localize beta oscillations source to the dorsal STN. Neurobiol Dis. 2020;146:105090. Epub 2020/09/26. doi: 10.1016/j.nbd.2020.105090. PubMed PMID: 32977021.

18.	Zaidel A, Spivak A, Grieb B, Bergman H, Israel Z. Subthalamic span of beta oscillations predicts deep brain stimulation efficacy for patients with Parkinson's disease. Brain. 2010;133(Pt 7):2007-21. Epub 2010/06/11. doi: 10.1093/brain/awq144. PubMed PMID: 20534648.

19.	van Wijk BCM, Alkemade A, Forstmann BU. Functional segregation and integration within the human subthalamic nucleus from a micro- and meso-level perspective. Cortex. 2020;131:103-13. Epub 2020/08/22. doi: 10.1016/j.cortex.2020.07.004. PubMed PMID: 32823130.

20.	Herz DM, Bange M, Gonzalez-Escamilla G, Auer M, Ashkan K, Fischer P, et al. Dynamic control of decision and movement speed in the human basal ganglia. Nat Commun. 2022;13(1):7530. doi: 10.1038/s41467-022-35121-8. PubMed PMID: 36476581; PubMed Central PMCID: PMCPMC9729212.

21.	Herz DM, Tan H, Brittain JS, Fischer P, Cheeran B, Green AL, et al. Distinct mechanisms mediate speed-accuracy adjustments in cortico-subthalamic networks. Elife. 2017;6. Epub 2017/02/01. doi: 10.7554/eLife.21481. PubMed PMID: 28137358; PubMed Central PMCID: PMCPMC5287713.
