# Supplementary material for: Dynamic modulation of subthalamic nucleus activity facilitates adaptive behavior
Source: PLoS Biol. 2023 Jun 1;21(6):e3002140. doi: 10.1371/journal.pbio.3002140 (PMC10234560; doi:10.1371/journal.pbio.3002140)
Supplement: Attachment — Submitted filename: ResponseToReviewers.docx [file pbio.3002140.s014.docx]

Reviewer remarks:

Reviewer #1: The authors addressed all questions. Congratulations to the study.

Reviewer #2: The authors did a great job answering all my questions. I have no further concerns.

Reply: We would like to thank the reviewers for the insightful comments and the positive evaluations.
